# Evaluation of Proteasome Inhibitors in the Treatment of Idiopathic Pulmonary Fibrosis

**DOI:** 10.3390/cells11091543

**Published:** 2022-05-04

**Authors:** I-Chen Chen, Yi-Ching Liu, Yen-Hsien Wu, Shih-Hsing Lo, Zen-Kong Dai, Jong-Hau Hsu, Yu-Hsin Tseng

**Affiliations:** 1 Department of Pediatrics, Kaohsiung Medical University Hospital, Kaohsiung Medical University, Kaohsiung 80756, Taiwan; yljane.chen@gmail.com (I.-C.C.); furtherchia@gmail.com (Y.-C.L.); eddiewu1986@gmail.com (Y.-H.W.); allenjay66@gmail.com (S.-H.L.); zenkong@gmail.com (Z.-K.D.); jhh936@yahoo.com.tw (J.-H.H.); 2 Department of Pediatrics, School of Medicine, College of Medicine, Kaohsiung Medical University, Kaohsiung 80708, Taiwan; 3 Graduate Institute of Medicine, College of Medicine, Kaohsiung Medical University, Kaohsiung 80708, Taiwan

**Keywords:** idiopathic pulmonary fibrosis, proteasome inhibitor, transforming growth factor-beta

## Abstract

Idiopathic pulmonary fibrosis (IPF) is the most common form of idiopathic interstitial pneumonia, and it has a worse prognosis than non-small cell lung cancer. The pathomechanism of IPF is not fully understood, but it has been suggested that repeated microinjuries of epithelial cells induce a wound healing response, during which fibroblasts differentiate into myofibroblasts. These activated myofibroblasts express α smooth muscle actin and release extracellular matrix to promote matrix deposition and tissue remodeling. Under physiological conditions, the remodeling process stops once wound healing is complete. However, in the lungs of IPF patients, myofibroblasts re-main active and deposit excess extracellular matrix. This leads to the destruction of alveolar tissue, the loss of lung elastic recoil, and a rapid decrease in lung function. Some evidence has indicated that proteasomal inhibition combats fibrosis by inhibiting the expressions of extracellular matrix proteins and metalloproteinases. However, the mechanisms by which proteasome inhibitors may protect against fibrosis are not known. This review summarizes the current research on proteasome inhibitors for pulmonary fibrosis, and provides a reference for whether proteasome inhibitors have the potential to become new drugs for the treatment of pulmonary fibrosis.

## 1. Introduction

Idiopathic pulmonary fibrosis (IPF) is a progressive, irreversible, and usually lethal disease characterized by an abnormal fibrotic response involving large areas of the lungs. Risk factors associated with IPF include smoking, environmental factors, comorbidities, and viral infections [1]. Most patients have persistent dyspnea and limited exercise tolerance resulting in a poor quality of life. Many patients develop pulmonary hypertension and are at an increased risk of pulmonary embolism and sudden cardiac death [2]. The molecular mechanisms underlying the pathogenesis and development of IPF are unclear, however molecules including chemokines, cytokines, growth factors, adenosine, glycosaminoglycans, and non-coding RNA, and cellular processes, including apoptosis, senescence, hypoxia, endothelial–mesenchymal transition, oxidative stress, mitochondrial dysfunction, endoplasmic reticulum stress, and alternative polyadenylation have been linked with the development of IPF [3]. Pirfenidone and nintedanib are the mainstays of current medical treatment of IPF, however they do not completely prevent or improve lung function. It is essential to find additional drugs that can effectively reduce the pro-fibrotic maturation of lung fibroblasts, and ultimately prevent IPF progression. Understanding the molecular mechanisms of IPF will aid in drug discovery. The wound healing response induced by repeated microinjuries of epithelial cells during which fibroblasts differentiate into myofibroblasts is a major contributor to IPF. Activated myofibroblasts express α smooth muscle actin (α-SMA) and release extracellular matrix (ECM) to promote matrix deposition and tissue remodeling [4,5]. In general, the remodeling process stops once wound healing is complete; however, myofibroblasts remain active and deposit excess ECM in the lungs of patients with IPF. This leads to the destruction of alveolar tissue, the loss of lung elastic recoil, and a rapid decrease in lung function. Several studies have suggested that proteasomal inhibition can decrease the expressions of ECM proteins and metalloproteinases. In addition, proteasome inhibitors have been reported to inhibit transforming growth factor (TGF)-β1-induced collagen I and tissue inhibitor of metalloproteinase-1 [6,7]. However, the mechanisms by which proteasomal inhibition may protect against fibrosis are not fully understood. This review summarizes the current research on proteasome inhibitors for pulmonary fibrosis, and provides a reference for whether proteasome inhibitors have the potential to become new drugs for the treatment of pulmonary fibrosis.

## 2. Risk Factors for Idiopathic Pulmonary Fibrosis

### 2.1. Intrinsic Risk Factors

Intrinsic risk factors including genetic susceptibility, aging, male sex, the lung microbiome, and comorbidities have been associated with the pathogenesis of IPF [8]. The susceptibility genes associated with the pathogenesis of IPF are currently classified into four categories: (1) genes related to alveolar stability (such as SFTPC, SFTPA1, SFTPA2); (2) genes related to accelerated cellular senescence by disrupting telomerase function (such as TERT, TERC, DKC1, PARN, and RTEL1); (3) genes related to host defense (such as MUC5B and TOLLIP); and (4) genes related to impaired integrity of the epithelial barrier (such as DSK) [8,9]. In addition, two genome-wide association studies reported that variants of MUC5B and TOLLIP are common [10,11]. 

IPF is also considered to be an age- and sex-related disease. IPF occurs mainly in elderly over 60 years of age, and the incidence and prevalence increase with age [12]. Germline mutations in telomerase (TERT) or its RNA component (TERC) are present in up to 10% of patients with IPF. Even in patients with IPF without a mutation in the telomerase gene, telomeres in peripheral blood leukocytes and in lung tissue have been reported to be shorter than those in controls [13,14]. Globally, IPF is more prevalent in men, possibly due to sex hormones. Several animal studies have indicated that male sex hormones are associated with accelerated fibrosis, and that female sex hormones may have a protective effect against pulmonary fibrosis [15,16]. However, the effects of sex hormones are organ- and species-specific, so sex hormone studies in humans are needed to determine their role in IPF. Testosterone is the most important male sex hormone. Plasma testosterone and leucocyte telomere length are significantly reduced, and testosterone is positively correlated with leucocyte telomere length in male patients with IPF [17]. Estrogen is a female sex hormone and may also contribute to the potential role of sex-specific differences in the lung. For example, TGF-β1 (a central factor in the development of pulmonary fibrosis) inhibits the expression of estrogen receptors, especially estrogen receptor alpha (ESR1) in human bronchial epithelial cells. In addition, TGF-β1 and estrogen inversely regulated the expression of several genes participating processes, such as extracellular matrix renewal, airway smooth muscle cell contraction, and calcium flux regulation [18].

The microbiome refers to the symbiotic and pathogenic microorganisms that make up the microbial ecosystem, and it has gained attention for its potential association with the initiation, perpetuation, and exacerbation of the fibrotic process in IPF [19]. Patients with IPF had a greater bacterial burden in bronchoalveolar lavage fluid (BALF) compared to controls and patients with moderate chronic obstructive pulmonary disease (COPD) [8,20]. Compared with healthy individuals, the microbiome of patients with IPF is enriched in *Haemophilus*, *Streptococcus*, *Neisseiria*, and *Veillonella* genera, which may play a causative role in acute exacerbation of IPF. Bacteria can cause epithelial alveolar injury and activate an immune cascade response due to their presence alone, the following pro-inflammatory and pro-fibrotic cascades leading to changes in lung architecture [21]. Greater bacterial burden in patients with IPF may be a biomarker for rapidly progressive disease and predicts worse survival [22]. In addition, mutations in the gene encoding MUC5B, which is essential for mucociliary clearance and in host-bacterial defense, have been associated with an increased incidence of IPF [23]. 

Common comorbidities in patients with IPF include gastroesophageal reflux (GER), obstructive sleep apnea, diabetes mellitus (DM), and herpesvirus infection. Repetitive lung injury from GER with subsequent secondary and chronic microaspiration has been considered as a risk factor in the pathogenesis of IPF. The prevalence of hiatal hernia on CT scan indicate that GER-related hiatal hernias occur more frequently in patients with IPF than in those with asthma or COPD [24]. The effect of anti-acid therapy on lung function changes are inconsistent [25,26], but the use of anti-acid therapy has been shown to be associated with longer survival. In addition, laparoscopic anti-reflux surgery may provide benefit on lung function of patients with IPF [27].

Obstructive sleep apnea is characterized by periodic apneas or hypopneas due to repetitive collapse of the upper airway during sleep. Despite the proximal occlusion, the respiratory muscles continue to make efforts to inspiration, so that the pleural pressure fluctuates greatly, resulting in traction microinjuries to the alveoli. These injuries result in aberrant epithelial cell activation, which, in combination with fibroblast recruitment, is involved in the pathogenesis mechanisms of IPF. [28,29].

Although definitive impacts of DM on the lungs is unclear, several studies have focused on the relationship between DM and pulmonary fibrosis. The hyperglycemia-mediated overproduction of advanced glycation end products leading to oxidative injury, and the subsequent overexpression of pro-fibrotic cytokines, fibroblast proliferation, and ECM deposition have been suggested as potential mechanisms by which DM may be a risk factor for IPF [30]. Metformin is the most commonly used oral diabetes medications, and it has been demonstrated to attenuate TGF-β1-mediated epithelial-mesenchymal transition (EMT) in vitro. In addition, metformin was also demonstrated to attenuate and reverse fibrosis in the bleomycin mouse model of pulmonary fibrosis [31,32]. 

Chronic viral infection, especially with members of the Herpesviridae family, cause repetitive alveolar epithelial injuries leading to the dysregulation of repair-responses, which has been proposed to be a mechanism of pulmonary fibrosis in IPF [33]. There are a greater proportion of Herpesviridae viruses have been identified in lung tissue and serum from subjects with IPF as compared to control subjects [34,35].

### 2.2. Extrinsic Risk Factors

As with other lung diseases, cigarette smoking is closely related to IPF. However, the mechanisms by which smoking affects the onset and progression of IPF are not fully understood. Cigarette smoking has been demonstrated to stimulate the overexpression of genes associated with EMT and a fibroblast-like phenotype in vitro [36], acceleration of telomere shortening in vivo [37], endoplasmic reticulum stress [38], and repetitive mechanical stretch [3,8]. Nicotine, the main chemical in tobacco, has addictive properties and can itself induce the production of TGF-β, an important mediator of fibrosis in IPF [39]. Consistent evidence has confirmed that cigarette smoking is associated with an increased rate of lung function loss, and that long-term smoking is an independent factor for IPF development. Moreover, IPF patients with a history of smoking have been reported to have shorter survival compared to those who have never smoked [3].

In addition, certain occupational and environmental exposure to pollutants may be associated with IPF. Some of the most common occupations involve exposure to such pollutants, including metallurgy, farming, textile work, welding, and veterinarians. For example, analyses of autopsy results from the United Kingdom [40] and Japan [41] found that metal workers had a relatively high risk of death from IPF. In addition, studies in Sweden and the United States have reported a direct relationship between exposure to wood dust and the risk of IPF. However, a significant number of IPF patients do not have any history of occupational exposure to pollutants. Some environmental factors, including dust, fibers, fumes, and particulate matter, may also contribute to the pathogenesis of IPF [3].

## 3. Mechanisms of Pulmonary Fibrosis 

Pulmonary fibrosis is an end-stage pathological change caused by chronic repetitive alveolar injuries of various causes (such as heredity, infection, and environmental exposure), resulting in excessive ECM deposition and accumulation. In contrast to pulmonary fibrosis induced by drugs, viral infection, or acute lung injury which may be partially stabilized and reversed after treatment, IPF is persistent and irreversible even after aggressive treatment [42,43]. After the hazard has been eliminated, reversible pulmonary fibrosis gradually resolves with treatment. Almost all animal models of pulmonary fibrosis are characterized by spontaneous regression. Bleomycin-induced pulmonary fibrosis animal models have the advantages of simple modeling method, low cost, and obvious fibrotic lesions, and so they are widely used in research [44]. In general, 28 days after bleomycin injury, the fibrotic lesions gradually regress and eventually approach normal [45]. IPF is the most common progressive pulmonary fibrosis disease and is considered to be absolutely irreversible. Due to the unclear pathogenesis and lack of relevant animal models, it is difficult to elucidate the causes and mechanisms by which irreversible pulmonary fibrosis occurs. Recently, researchers showed that repetitive intratracheal instillation of bleomycin in young mice or a single dose of bleomycin in aged mice resulted in persistent pulmonary fibrosis without spontaneous resolution, and these models can provide the basis for pathogenesis studies on persistent pulmonary fibrosis [46,47,48]. 

**Figure 1:**
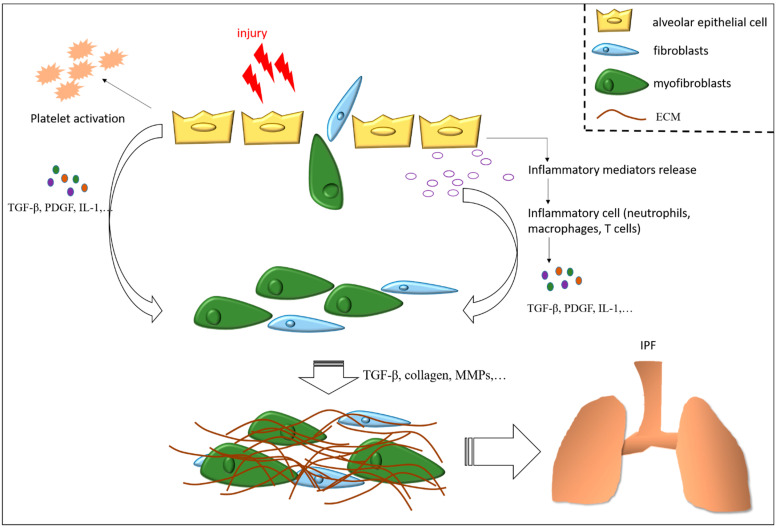
Overview of wound healing leading to the development of fibrosis. Epithelial cell injury induces the secretion of inflammatory mediators and triggers platelet activation, thereby increasing vascular permeability and the recruitment of leukocytes. These inflammatory cells release pro-fibrotic cytokines, such as TGF-β, which mediate activation and recruitment of fibroblasts, differentiation of myofibroblasts, and release of ECM components, thereby promoting wound healing. Abnormal wound repair responses lead to the irreversible and excessive scar tissue within the lungs of patients with IPF.

The pathogenesis of persistent pulmonary fibrosis involves a complex network. Lung injury induces fibroblast recruitment, leading to collagen deposition and fibrosis. In addition, abnormal alveolar epithelial hyperplasia and the overproduction of mucin due to the incomplete differentiation of alveolar epithelia, which may cause interference with wound healing and promote pulmonary fibrosis. The loss of the endothelial phenotype and high vascular permeability cause pulmonary vascular dysfunction, which may induce abnormal vascular remodeling and further enlarges the fibrotic lesions, and then lead to the persistent and progressive development of pulmonary fibrosis. Although the pathogenesis of irreversible pulmonary fibrosis is unclear, factors associated with the development of fibrosis, including apoptosis resistance of (myo)fibroblasts, dysfunction of pulmonary vessels, cell mitochondria and autophagy, aberrant epithelial hyperplasia, and lipid metabolism disorder have been reported [48]. The wound repair process can be dysregulated in any stage of fibrosis associated with IPF. The most significant wound healing stages leading to the development of pulmonary fibrosis are represented in Figure 1.

### 3.1. Apoptosis Resistance of (Myo)Fibroblasts

In the process of wound healing, fibroblasts are recruited to the injured area by epithelial injury-induced inflammation and differentiate into myofibroblasts induced by TGF-β. Normally, myofibroblasts gradually undergo apoptosis as the wound heals. However, in pathological conditions, persistent activation of (myo)fibroblasts leads to excessive scar hyperplasia and organ fibrosis [49]. Altered levels of apoptosis resistance in IPF (myo)fibroblasts lead to their persistent activation and pulmonary fibrosis [50]. In the fibrotic lung, elevated levels of reactive oxygen species (ROS)-related factor NADPH oxidase 4 (Nox4) induce lung fibroblasts to transform into a senescent and apoptosis-resistant phenotype, promoting pulmonary fibrosis. The expression of Nox4 has been reported to be significantly increased, and the expression of Nrf2, an antioxidant factor that can neutralize Nox4, to be significantly decreased in lung fibroblasts from patients with IPF and bleomycin-induced pulmonary fibrosis mice [51]. In IPF, dysfunction of death factor Fas signaling induces lung fibroblasts which are resistant to apoptosis and retain the pro-fibrotic phenotype and persistently activate COL1A1 and α-SMA promoters [52]. 

### 3.2. Dysfunction of Pulmonary Vessels

Pulmonary vessels are responsible for carrying blood for gas exchange and nutrient transport in a mature lung. In addition, pulmonary capillary endothelial cells (PCECs) release various cytokines to support the development, regeneration, and wound healing of the lungs. An imbalance in the abundance of pulmonary vascular endothelial cells and progenitors, as well as an imbalance between profibrotic and antifibrotic cytokines may result in aberrant vascular remodeling and alveolar capillary permeability changes. The degree of increased vascular permeability has been associated with the prognosis of patients with IPF [48,53]. 

Following lung injury, endothelial cells increase the expression of nitric oxide synthase 3 (NOS3) to synthesize endothelial nitric oxide synthase, which causes nitric oxide activate soluble guanylate cyclase, thereby promoting inactivation of lung fibroblasts and regression of pulmonary fibrosis [54]. Lung injury induce the activation of PCECs and the expression of chemokine receptor CXCR7, which protects alveolar epithelial cells from injury by inhibiting Jag1-Notch pathway-mediated EMT and pulmonary fibrosis. However, the degeneration of pulmonary vessels causes the reduction in vessel density, the loss of the endothelial phenotype and unable to encode NOS3 by endothelial cells, thereby resulting in persistent pulmonary fibrosis. Chronic lung injury caused by repetitive bleomycin instillation has been shown to suppress the expression of CXCR7 and promote the recruitment of macrophages around vessels [48,55], which stimulates PCECs to increase Wnt/β-catenin-dependent Jag 1 (one Notch ligand), thereby promoting persistent pulmonary fibrosis through the sustained activation of Notch signaling in perivascular fibroblasts [48,55].

### 3.3. Mitochondrial Dysfunction

Mitochondrial dysfunction is considered an important pathological feature of pulmonary fibrosis. Peroxisome proliferator-activated receptor gamma co-activator 1-alpha (PGC1α) is a transcriptional coactivator. In addition to regulating mitochondrial biogenesis, oxidative phosphorylation, and ROS detoxification, PGC1α also mediates the regression of fibrotic lesions [56]. The stable inhibition of PGC1α has been demonstrated to reduce mitochondrial mass and function in IPF lung fibroblasts [57]. Mitochondrial dysfunction induces persistent pulmonary fibrosis through activating a pro-fibrotic fibroblast phenotype and promoting the senescence of adjacent cells via a paracrine mechanism. In addition, PTEN-induced putative kinase 1 (PINK1) is a key regulator of mitochondrial function, and is low expression in aged-related lungs and IPF lungs [48,58]. In addition, a lower expression of PINK1 has been shown to cause mitochondrial dysfunction in type II alveolar epithelial cells (ATIIs), leading to endoplasmic reticulum stress and mitophagy dysfunction. Furthermore, a deficient expression of PINK1 in ATIIs can induce the release of profibrotic factors. 

### 3.4. Autophagy Dysfunction

Autophagy is an important cytoprotective mechanism that can maintain cellular homeostasis and regulate redox balance. In fibroblasts and alveolar epithelia, decreased autophagy induces activation of lung fibroblasts and promotes pulmonary fibrosis. Moreover, autophagy dysfunction induces apoptosis-resistant lung fibroblasts and persistent pulmonary fibrosis by activating the mammalian target of rapamycin signaling pathway in IPF lung fibroblasts. In lung endothelial cells, impaired autophagic flux induces the changes of endothelial structure and affects the progression of pulmonary fibrosis, which may be accompanied by a loss of the autophagy gene ATG7 [59,60].

### 3.5. Aberrant Epithelia Hyperplasia and Dysfunction

The alveolar epithelium is composed of type I alveolar epithelial cells (ATIs) and ATIIs [61]. Histological analysis showed that more ATIIs in the lungs of patients with IPF or bleomycin-induced pulmonary fibrosis mice compared with controls, particularly prominent in areas close to fibrobastic foci consisting of small dome-shaped collections of spindle-forming (myo)fibroblasts within a myxoid-appearing matrix [62]. Specimens from the control group showed normal alveolar characteristics similar to ATIs and ATIIs lined with thin-walled alveolar septa. Specimens from patients with IPF present with the pathological pattern of usual interstitial pneumonia (UIP), including patchy fibrosis and architectural distortion and fibroblast foci [63]. In normal lungs, ATIs cover over 90% of the alveolar surface. During lung injury, ATIs are susceptible to damage and even death. ATIIs are regarded as stem cells of the alveolar epithelium to participate alveolar epithelial repair [61,64]. Lung injury induces the activation and proliferation of surfactant-producing ATIIs to form wound clots, which are constructed by hyperplasia of ATIIs covering exposed alveolar surfaces, the activation of local coagulation pathways, and initiation of provisional matrix formation [65]. Hyperplastic ATIIs regulate apoptosis and have the ability to transdifferentiate into ATIs to re-establish a fully functional alveolar epithelium [66]. During normal wound healing, lung tissue eventually returns to its original structure and function as the provisional matrix gradually dissipates. However, persistent disturbance of the epithelial basement membrane following extensive damage may lead to alveolar collapse and ATIIs fail to re-epithelialize [67]. This results in the initiation of an abnormal wound repair response, whereby epithelial cells, mainly ATIIs, release pro-fibrotic cytokines, growth factors, and other chemokines at the site of injury to promote the activation and proliferation of (myo)fibroblasts and to increase ECM stiffness in IPF [68,69].

### 3.6. Lipid Metabolism Disorder

The balance of lipid metabolism is critical for maintaining the structure and function of the alveolar epithelium. Excessive accumulation of cholesterol leads to alveolar collapse and injury [70]. Elongation of long-chain fatty acids family member 6 and stearoyl CoA desaturase 1 are lipid metabolism-related molecules, and levels of these molecules have been reported to be reduced in IPF lungs [71,72]. In addition, suppression of these genes in mice has been shown to increase fibrosis susceptibility. Sequencing data have revealed that the genes and signaling pathways related to lipid metabolism are down-regulated in the lungs of IPF patients and in aged mice with bleomycin injury [73,74].

### 3.7. Transforming Growth Factor-Beta in Idiopathic Pulmonary Fibrosis

Growth factors, such as TGF-β, insulin-like growth factor-1 (IGF-1), binding proteins, tumor necrosis factor-alpha (TNF-α), platelet-derived growth factor (PDGF), interleukins (ILs), endothelin-1, connective tissue growth factor (CTGF), vascular endothelial growth factor (VEGF), and fibroblast growth factor (FGF) have been shown to be involved in the pathology of IPF at a molecular level. Of these factors, TGF-β has been identified as a central factor in the development of pulmonary fibrosis [3]. Alveolar epithelial damage leads to the recruitment of fibroblasts, which are activated by TGF-β, resulting in collagen deposition and organ fibrosis. TGF-β is a superfamily of more than 35 structurally different protein isoforms, of which TGFβ-1, TGFβ-2, and TGFβ-3 are present only in mammals and are known to act as major pro-fibrotic factors in the pathogenesis of fibrosis through multiple pathways, and to exhibit different phenotypes and functions [2,75]. TGF-β was named after the discovery of the protein TGFβ-1, which is highly expressed in IPF [6]. In fibrosis, TGF-β has both stimulatory and inhibitory properties. TGFβ-1 is involved in the promotion and induction of fibrosis in various tissues. In addition, TGF-β1 is the only isoform of TGF-β to affect the function of the endocrine system. In IPF, TGF-β acts as a pro-fibrotic factor in the process of EMT through both Smad-dependent and Smad-independent pathways. If TGF-β is activated through the Smad-dependent pathway, it affects the genetic level of α-SMA, collagen, and PAI-1 [76]. In addition, TGF-β causes the upregulation of IGF in fibrotic tissue and fibroblast cells, resulting in altered lung function. TGF-β is a pleiotropic cytokine which damages lung tissue, and plays a role in lung tissue development and in maintaining homeostasis in other tissues of the lungs [77,78]. 

### 3.8. Inflammation

Inflammation and changes in innate and adaptive immune responses have also been implicated in the development of IPF. Inflammatory cells in the lungs of patients with IPF have been shown to produce increased levels of ROS, which can drive the production of pro-inflammatory cytokine, including IL-1β [67]. The secretion of IL-1β has been associated with the progression of fibrosis by enhancing the expressions of the inflammatory mediators IL-6 and TNF-α, disrupting alveolar structure, and increasing lung fibroblasts, as well as collagen deposition [79]. In addition, IL-1β has been shown to increase lung infiltration by neutrophils and macrophages, and to increase the expressions of matrix metalloproteinase and chemokine ligands [80]. IL-1β in BALF has also been shown to stimulate the release of the pro-fibrotic cytokines TGF-1 and PDGF [79].

The pro-inflammatory cytokine IL-17A is expressed by CD4^+^ T-helper (T_H_-17) cells, and has been linked with enhanced the recruitment of neutrophil and TGF- and IL-1β-mediated fibrosis [81]. In addition, increased percentage of neutrophil in BALF is considered to be a prognostic predictor of early mortality in patients with IPF [82]. T_H_-1 effector T cells are thought to induce anti-fibrotic activities through the production of interferon-γ [83], and T_H_-2 effector T cells are thought to promote fibrosis through the production of some cytokines, such as IL-4, IL-5, and IL-13 [84,85].

## 4. The Mainstay of Medication and The Potential of Proteasome Inhibitors for IPF

Pirfenidone and nintedanib were approved by the FDA for the treatment of IPF in 2014 based on positive phase 3 trials, and they are currently the mainstay of medical therapy for IPF, with acceptable safety and tolerability. With the approval of pirfenidone and nintedanib for patients with mild-to-moderate IPF, early diagnosis is a prerequisite for earlier treatment. These drugs help to prevent further scarring and slow the progression of the disease, but do not cure IPF. In addition, there are insufficient data on proven effective treatments for severe IPF, although it may also be because patients with severe IPF usually not participate in randomized, prospective, multicenter, international trials [86]. It is necessary to find novel effective treatment strategy for IPF. New drugs and combinations of pirfenidone or nintedanib with other drugs have subsequently been developed. For example, the autotaxin-lysophosphatidic acid (ATX/LPA) pathway, CTGF, pentraxin-2, G protein-coupled receptor agonists/antagonists, αvβ6 integrin, and galectin-3 are novel targets that have been shown to be effective in phase 2 clinical trials [18]. In addition, several studies have indicated that proteasome inhibition can provide anti-fibrotic effects in different tissues and in several experimental mouse models. However, the effect of proteasome inhibitors on pulmonary fibrosis remains controversial. The mechanisms mediating these anti-fibrotic effects have yet to be fully elucidated, however they appear to involve the attenuation of pro-fibrotic TGF-β signaling. In the following sections, we review relevant studies on the effect of proteosome inhibitors on pulmonary fibrosis, and evaluate the potential of using proteasome inhibitors in the treatment of pulmonary fibrosis. 

A number of different treatments, with advantages and disadvantages, have been used to induce pulmonary fibrosis in animals. Although none of them induce the same pathology as human IPF, each model recapitulates some key features of IPF and provide a convenient platform to study collagen regulation in disease settings. Agents for inducing pulmonary fibrosis in animals include etiologic agents (such as asbestos, silica, and radiation) and chemical agents (such as bleomycin, monocrotaline, fluorescein isothiocyanate, oxidants, and phorbol myristate acetate). The bleomycin-induced lung fibrosis model is the most widely used [87]. Bleomycin is an anticancer drug that induces DNA damage in target cells, and is usually administered as a single dose in saline or PBS by intratracheal, intranasal, intraperitoneal, oropharyngeal, or intravenous routes. A continuous or repetitive delivery method of bleomycin induce more fibrosis in the lung, and fibrotic phenotype more similar to IPF than single bleomycin delivery methods [47]. Selecting an appropriate mouse strain is important because there is strong evidence that genetic background can influence the degree of pulmonary fibrosis following bleomycin treatment. C57BL/6J mice are the most commonly used strain for bleomycin treatment, because of the reproducibly high levels of inducible lung collagen deposition for at least 12 weeks [88].

### 4.1. Pirfenidone and Nintedanib

Pirfenidone is an anti-fibrotic and anti-inflammatory drug which reduces fibroblast proliferation and the accumulation of collagen [89]. Pirfenidone should be taken three times daily with meals, with a target dose of 801 mg, which is usually achieved within two weeks. The details are a dose of 267 mg administered three times a day (801 mg/day) for 1 week, a dose of 534 mg administered three times a day (1602 mg/day) for 1 week, and then a dose of 801 mg administered three times daily thereafter (2403 mg/day). Baseline liver enzyme levels should be measured prior to taking pirfenidone, and subsequently monitored at monthly for 6 months, and then every 3 months thereafter. Pirfenidone should not be administered to patients with Child-Pugh Class C hepatic impairment or those requiring dialysis [90,91]. Side effects of pirfenidone include rash, photosensitivity, and gastrointestinal discomfort. Therefore, patients taking pirfenidone are advised to avoid exposure to sunlight or use sunscreen and clothing to protect from sun exposure. In addition, if gastrointestinal symptoms persist with pirfenidone with meals, antacids and antiemetics may be prescribed. However, omeprazole may modulate pirfenidone level, so omeprazole treatment should be avoided in patients taking pirfenidone. If side effect symptoms or hepatotoxicity occur, the dose can be reduced, or temporarily discontinued and then reintroduced after a few weeks using a slower dose titration. [92]. Because pirfenidone is mainly metabolized by cytochrome P450 1A2 (CYP1A2) enzymes, patients should avoid the concomitant use of other CYP1A2 inhibitors (e.g., fluvoxamine and ciprofloxacin) or inducers (e.g., tobacco, omeprazole, and rifampicin) [93].

Nintedanib is a small-molecule inhibitor of receptor tyrosine kinases, including FGF receptor, PDGF receptor, and VEGF receptor [94]. Nintedanib should be taken orally 150 mg twice daily, and liver enzymes should be monitored monthly for 3 months and every 3 months thereafter. In addition, nintedanib treatment is not recommended for patients with moderate or severe liver impairment (Child-Pugh Class B or C) [90]. Side effects of nintedanib include diarrhea and nausea, which can often be effectively controlled with antidiarrheal medications or antiemetics [95,96]. As with pirfenidone treatment, when side effect symptoms or hepatotoxicity occur, the dose can be reduced, or temporarily discontinue and reintroduce after a few weeks using a slower dose titration. Since nintedanib is a substrate of P-glycoprotein (P-gp) and CYP3A4, the co-administration with oral doses of P-gp and CYP3A4 inhibitors (e.g., ketoconazole and erythromycin) should be avoided, so as not to increase exposure to nintedanib [97].

Multiple trials have demonstrated that both pirfenidone and nintedanib were associated with a reduction in mortality compared to placebo, and could effectively reduce lung volume loss, regardless of the initial reported forced vital capacity and diffusing capacity of the lung for carbon monoxide [98,99]. Of note, nintedanib may increase the risk of bleeding by inhibiting VEGF receptor signal transduction, so pirfenidone may be a better option than nintedanib when patients receive full-dose anticoagulation or dual antiplatelet therapy [94,100]. However, the higher proportion of bleeding in clinical trials of patients taking nintedanib compared to placebo were minor events, such as bruising or epistaxis. Therefore, nintedanib may be selected over pirfenidone based on a patient’s inability to avoid sun exposure, and in those with pre-existing dermatologic conditions. The co-administration of nintedanib and pirfenidone has been shown to have a manageable safety and tolerability profile in patients with IPF, with no relevant effects on pharmacokinetic drug–drug interactions [90,101,102]. However, clinical trials are still needed to assess whether this combination can improve efficacy.

### 4.2. Overview of Proteasome Inhibitors and the Effects of Proteasome Inhibitors in Patients with Pulmonary Fibrosis

The ubiquitin-proteasome system is responsible for the programmed degradation of most intracellular proteins. Proteins are targeted for proteasomal degradation by linkage to polyubiquitin chains as a degradation signal. Polyubiquitination proceeds along a cascade of enzymatic reactions involving E1, E2, and E3 enzymes which transfer activated ubiquitin to lysine residues of substrate proteins. The polyubiquitinated proteins are then transferred to and hydrolyzed by proteasomes [103,104]. Proteasomes are multimeric protease complexes and the center of cellular protein degradation, thereby activating or shutting down some pathways. The 26S proteasome is a multicatalytic enzyme complex, comprising a 20S core catalytic complex with 19S regulatory subunits at each end. The 20S core catalytic complex contains three active sites residing in the β5, β2, and β1 subunits that cleave polypeptides after different amino acids, which are named chymotrypsin-like (CT-L), trypsin-like (T-L), and caspase-like (C-L) active sites, respectively [105]. Proteasomes are present in all cells, but they are relatively abundant in multiple myeloma cells, making that disease a target for proteasome inhibitors. Many proteasome inhibitors are currently in development. Although several protease inhibitors are developed, the molecular mechanism has not been fully studied. These compounds have commonly been reported to be inhibitors of the NF-κB pathway. However, the regulation of the NF-κB pathway by the same proteasome inhibitor may still vary depending on the cell type. In addition, proteasome inhibitors have been reported to induce apoptosis by regulating pathways other than NF-κB. These compounds incorporate different chemical warheads to inhibit the catalytic activity of the proteasome. Currently, proteasome inhibitors used in research or clinical treatment for multiple myeloma were listed in Table 1. Several studies are also actively investigating the effectiveness of proteasome inhibitors in various diseases including pulmonary fibrosis. 

#### 4.2.1. MG-132

The first synthetic proteasome inhibitor contained a peptide backbone with an aldehyde on its C terminus forming a reversible complex with the active site threonine. MG-132, originally named ZLLLal, is a modified version with a different peptide backbone, and it is more potent and cell permeable [106].

Studies indicated that MG-132 suppress NF-κB activity in various cells by inducing nuclear translocation and accumulation of IκBα, which then binds with NF-κB p50/65 and interferes DNA binding activity of NF-κB [107,108]. The combination therapy of MG-132 and GSK-470, a PDK1 inhibitor, induce apoptosis by inhibiting phosphorylation of mTOR and AKT, and inducing nuclear accumulation of PTEN in multiple myeloma cells [109].

Myocardial remodeling is an adaptive response of the myocardium to several forms of stress, ultimately leading to cardiac fibrosis, left ventricular dilation, and loss of contractility. MG-132 has been shown to suppress the activity of matrix metalloproteinases (MMPs) and the RNA expressions of MMPs and collagens in rat cardiac fibroblasts. In addition, MG-132 treatment over 12 weeks was shown to effectively suppress the expressions of MMPs and collagens in spontaneously hypertensive rats, resulting in a marked reduction in cardiac fibrosis compared with control animals [6,110]. In NRK-49F cells (rat renal interstitial fibroblasts), MG-132 was shown to downregulate the expressions of CTGF, α-SMA, fibronectin and collagen III simulated by TGF-β1 [111].

Tank binding protein kinase-1 (TBK1) is a kinase that was recently identified as a candidate regulator of fibroblast activation. Reducing the activity or expression of TBK1 has been shown to result in a 40–60% reduction in smooth muscle actin stress fiber levels and a 50% reduction in the deposition of the ECM components collagen I and fibronectin in TGF-stimulated normal and IPF fibroblasts. In addition, yes-associated protein (YAP) and transcriptional coactivator with PDZ-binding motif (TAZ) are related mechanosensory proteins known to regulate fibroblast activation [112]. TBK1 stabilizes YAP/TAZ levels by reducing YAP/TAZ proteasome degradation, and TBK1 knockdown or inhibition has been shown to reduce the total and nuclear protein levels of YAP/TAZ. The treatment of fibroblasts with MG-132 has been shown to result in increased YAP/TAZ levels in both TBK1 siRNA and non-targeting siRNA control-treated cells [113]. These results suggest that proteasome inhibitors may promote fibroblast activation by reducing YAP/TAZ proteasome degradation.

#### 4.2.2. Bortezomib

Bortezomib, a dipeptide boronic acid derivative, is the first proteasome inhibitor to receive FDA approval for the treatment of multiple myeloma and other plasma cell malignancies, and it has been associated with significant improvements in response rates and overall survival in front-line and relapsed/refractory settings [104,114]. Numerous clinical trials of bortezomib focusing on its efficacy in other tumors, in combination with other drugs, and for non-cancer applications have also been conducted [106]. Bortezomib is formulated as a mannitol ester and administered via intravenous or subcutaneous routes. Bortezomib is rapidly cleared from the body; however, accumulation occurs after repeated dosing [115]. 

Many studies indicated that bortezomib stabilized the inhibitor IκB in cytosol leading to reduced activation of NF-κB [116,117]. Bortezomib also inhibits cyclin turnover, which affects cyclin-dependent kinase (CDK) activity. This may be particularly relevant to the action of bortezomib for treating mantle cell lymphoma, as which often caused by a gene translocation that causes the overexpression of cyclin D1 [118]. In addition, bortezomib affects telomerase activity [119], kinases such as JNK, tumor suppressors such as p53, and the Bcl-2 family of proteins has been reported [106].

Several studies have investigated the potential of bortezomib for the treatment of fibrosis. In a mouse study of bile duct ligation-induced cirrhosis, a single dose of bortezomib was given three days after bile duct ligation, which significantly reduced the expressions of α-SMA and collagen, and attenuated the severity of histological fibrosis [120]. In a murine model of thrombopoietin-induced myelofibrosis, reduced levels of TGF-β1 in bone marrow fluid and impaired development of spleen fibrosis after bortezomib treatment for four weeks have also been demonstrated. Moreover, bortezomib treatment impaired the development of osteosclerosis and increased one year survival rate from 8 to 89% after 12 weeks of treatment [121]. In both human dermal fibroblasts and murine fibroblasts, bortezomib has been shown to reduce collagen I mRNA expression [7]. In addition, bortezomib effectively inhibit TGF-β1-mediated target gene expression by inhibiting Smads activated transcription in primary human lung fibroblasts from normal individuals and patients with IPF, and in skin fibroblasts from patients with scleroderma. This response is due to increased abundance and activity of peroxisome proliferator activated receptor γ, a Smad-mediated transcriptional repressor [122]. In addition, bortezomib inhibits the pro-fibrotic activity induced by BALF from patients with pulmonary fibrosis. Notably, bortezomib treatment is effective even with bleomycin-induced acute lung injury peaking and TGF-β1 activation in the lung, which differs from other therapeutic strategies shown to inhibit bleomycin-induced pulmonary fibrosis [122]. 

However, some studies have raised concerns. For example, bortezomib has been shown to inhibit the chymotryptic activity of proteasomes but to enhance JNK and TGF-β signaling, which has been shown to promote fibrosis in vivo. Moreover, bortezomib failed to prevent bleomycin-induced lung inflammation and fibrosis or attenuate skin fibrosis in TSK-1/1 mice [7]. Furthermore, another study indicated that the therapeutic administration of bortezomib could diminish the severity of pulmonary fibrosis, and that this effect was independent of proteasome inhibition, and rather attributable to the induction of dual-specificity protein phosphatase 1 [123]. 

#### 4.2.3. Carfilzomib

Carfilzomib, originally derived from the naturally occurring epoxomicin, received initial FDA approval for relapsed and refractory myeloma in 2012, and it is the only approved drug with a reactive epoxide pharmacophore, a feature previously considered unsuitable for drug development. Carfilzomib, such as bortezomib, is administered intravenously and is a useful treatment to overcome some forms of bortezomib resistance [124]. Carfilzomib has been demonstrated to have fewer off-target effects and stronger proteasome inhibition effects relative to bortezomib [125,126]. In addition, the reported rates of peripheral neuropathy are >60% lower in patients receiving carfilzomib compared to those receiving bortezomib. 

In chronic lymphocytic leukemia cells, carfilzomib potently induces apoptosis by caspase-dependent and occurs irrespective of p53 status. In addition, carfilzomib promotes atypical activation of NF-κB, which is manifested by loss of cytoplasmic IkBα, phosphorylation of IκBα and DNA binding of NF-κB p50/p65, without subsequent increases in canonical NF-κB target gene transcription [127].

#### 4.2.4. Oprozomib

Oprozomib is a truncated derivative of carfilzomib, and it is the first orally bioavailable epoxyketone-based proteasome inhibitor. Orally bioavailable proteasome inhibitors could allow for more flexible dosing and be more convenient for patients. In addition, oral oprozomib was shown to delay tumor growth in a myeloma xenograft with efficacy similar to intravenous carfilzomib [128]. Both oprozomib and carfilzomib have been shown to inhibit the chymotrypsin-like activity of proteasomes and induce cell death in myeloma cell lines and primary cells from patients. In addition, oprozomib has been shown to decrease the viability of multiple myeloma cell lines and primary tumor cells from patients without affecting the viability of normal hematopoietic cells [124].

One study reported that local lung-specific treatment with oprozomib resulted in an antifibrotic effect without systemic toxicity in a mouse model of pulmonary fibrosis. Oprozomib was less toxic than bortezomib and was highly selective for the chymotrypsin-like active site of proteasomes. In addition, oprozomib treatment eliminated the expression of collagen-I and α-SMA induced by TGF-β in primary mouse lung fibroblasts. However, locally applied oprozomib failed to reduce fibrosis in bleomycin-challenged mice, and resulted in accelerated weight loss and increased mortality [105]. Specifically targeting activated proteasome complexes in the fibrotic lung to the right degree and at the right time point may be necessary for the treatment of pulmonary fibrosis with proteasome inhibitors.

#### 4.2.5. Ixazomib

Ixazomib was the first oral proteasome inhibitor approved by the FDA for the treatment of relapsed multiple myeloma in 2015 [125]. It preferentially inhibits CT-L activities of the 20S proteasome, with 10- and 1000-fold less potency for C-L and T-L activities, respectively. Ixazomib is similar in selectivity and potency to bortezomib, however the proteasome binding kinetics of these two molecules are different. Both ixazomib and bortezomib exhibit time-dependent reversible proteasome inhibition, however, the proteasome dissociation half-life (t1/2) of ixazomib is about 6-fold faster than that of bortezomib (t1/2 of 18 and 110 min, respectively) [129]. In addition, preliminary pharmacokinetic results indicate that the fixed-dose administration of ixazomib is feasible, making oral administration of the drug very convenient.

#### 4.2.6. Delanzomib

As with bortezomib, delanzomib is a reversible proteasome inhibitor of the peptide boronic acid class, and it exhibits similar potency against proteolytic activities of proteasomes in human erythrocytes, multiple myeloma, and HeLa cancer cells. However, delanzomib has been shown to be active as an oral formulation in preclinical studies, and delanzomib has shown greater and more sustained dose-related inhibition of tumor proteasome activity than bortezomib following the maximum tolerated dose of bortezomib or delanzomib in severe combined immunodeficiency mice. In addition, delanzomib has shown similar or better single-agent antitumor activity in primary multiple myeloma plasma cells in vitro compared to bortezomib [130].

#### 4.2.7. Marizomib

Marizomib (NPI-0052, Salinosporamide A) is an orally active, small molecule proteasome inhibitor derived from Salinospora tropica (marine actinomycete bacteria) [131]. Unlike other peptide-based proteasome inhibitors, marizomib has a β-lactone-γ-lactam bicyclic ring structure without a linear peptide backbone. In multiple myeloma cells and purified proteasomes, marizomib has been shown to irreversibly inhibit proteasome activity at nanomolar concentrations [132]. In addition, marizomib can target proteasomes more broadly, as it inhibits all three major proteolytic activities (preferentially inhibiting CT-L activity, followed by T-L activity and to a much lesser extent C-L activity) [133,134,135]. Marizomib has been tested in phase I, II, and III clinical trials in a variety of cancers, including refractory multiple myeloma, leukemia, lymphoma, glioblastoma, and malignant glioma. The results from these trials have shown that marizomib either as monotherapy or in combination with pomalidomide is well-tolerated and demonstrates promising activity in relapsed and refractory multiple myeloma [131].

Although the molecular mechanism of newly developed next generation of proteasome inhibitors still needs to be investigated. It has been reported that bortezomin, carfilzomib, and marizomib can inhibited the activity of non-canonical NF-κB signaling pathway and induced the apoptosis in cytarabine-resistant HL60 Cells [136]. Bortezomin and marizomib decrease the viability of pulmonary arterial smooth muscle cells by restoring mitofusin-2 expression under hypoxic conditions [137]. 

## 5. Challenges in the Treatment of Pulmonary Fibrosis with Proteasome Inhibitors

Peripheral neuropathy is a common and often dose-limiting toxic side effect of many active chemotherapeutic agents [138]. The National Cancer Institute lists chemotherapy-induced peripheral neuropathy as a reason for discontinuing treatment [139]. Peripheral neuropathy refers to damage, inflammation, or degeneration of peripheral nerves. The main symptoms of peripheral neuropathy are numbness, tingling, paresthesia, dysesthesia, pain, and weakness [140]. Bortezomib has been reported to induce autonomic peripheral neuropathy, causing neuropathic pain, orthostatic hypotension, bradycardia, sexual dysfunction, and constipation. Therefore, low toxicity is an important requirement for the development of the next generation of proteasome inhibitors [140,141]. 

In addition, proteasome inhibitors targeting a single active site have been shown to lead to compensatory activation of other active sites resulting in drug resistance, so that efficient inhibition of more than one active site is required to induce cell death [105,131,142]. The development of irreversible pan-proteasome inhibitors may be an effective way to overcome drug resistance. Currently, the only irreversible pan-proteasome inhibitor of T-L activity, CT-L activity, and C-L activity in development is marizomib [131], and clinical trials have indeed shown that marizomib is well-tolerated with promising activity in relapsed and refractory multiple myeloma [131,143]. 

**Figure 2:**
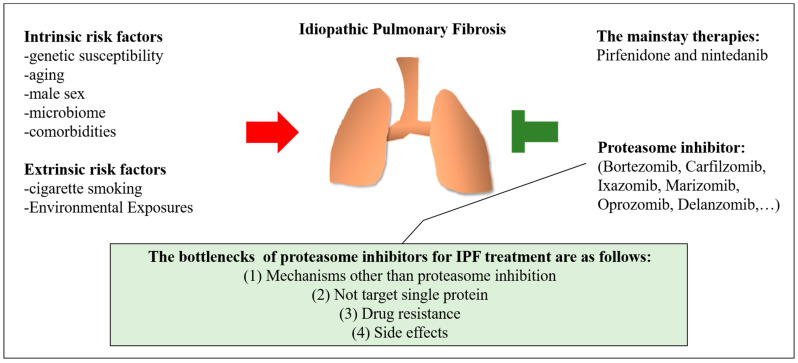
Overview of risk factors and treatments for idiopathic pulmonary fibrosis. The risk factors for idiopathic pulmonary fibrosis (IPF) include intrinsic risk factors (such as genetic susceptibility, aging, male sex, the lung microbiome, and comorbidities) and extrinsic risk factors (such as cigarette smoking and environmental exposure). Pirfenidone and nintedanib are the mainstay of medical therapy for IPF. Although the role of proteasome inhibitors in pulmonary fibrosis remains uncertain, they have been reported to potentially have anti-fibrotic effects.

In conclusion, current evidence indicates that proteasome inhibitors have anti-fibrosis effects, such as reducing fibroblast proliferation, differentiation into myofibroblasts, and collagen synthesis. However, the in vivo efficacy of proteasome inhibitors in pulmonary fibrosis and the dependence on proteasome inhibition have yet to be conclusively defined. Based on our review of the current research, the bottlenecks encountered in the use of proteasome inhibitors are as follows: (1) proteasome inhibitors cause anti-fibrosis effects through mechanisms other than proteosome inhibition [123]; (2) the activity of proteasome inhibitors leads to the accumulation of several proteins that are degraded by the proteasome machinery, but does not target a single protein [136]; (3) several proteasome inhibitors only inhibit one activity site of proteasomes, which causes compensatory activation of other activity sites, resulting in drug resistance [131,136]; and (4) toxicity of proteasome inhibitors causes side effects, making them difficult to apply in vivo [140,141]. At present, studies associated anti-fibrotic effect of proteasome inhibitors focus on fibroblasts, however, several studies demonstrated that the importance of proteasome function in maintaining ATIIs homeostasis [144,145]. In ATIIs of mice, partial deletion of RPT3, which promotes assembly of active 26S proteasome, leads to augmented cell stress and cell death. Acute loss of ATIIs resulted in alveolar surfactant depletion and alveolar epithelial barrier disruption leading to lethal acute respiratory distress syndrome [144]. These results point to the importance of proteasome function in maintaining ATIIs homeostasis and this issue requires attention in the development of proteasome inhibitor treatment for IPF. Understanding the complex mechanisms of proteasome inhibitors, developing irreversible pan-proteasome inhibitors, and reducing the toxicity of proteasome inhibitors are important issues that must be solved before proteasome inhibitors are used in the treatment of pulmonary fibrosis. An overview of risk factors and treatment for IPF is shown in Figure 2. 

## Figures and Tables

**Table 1:** Examples of proteasome inhibitor classes.

Compound	FDA Approval	Class	Effect	Activity	Administration
MG-132	just used in laboratories	peptide aldehydes	reversible	T-L, CT-L	N/A
Bortezomib	FDA approval in 2003	boronic acid	reversible	CT-L	IV, SC
Carfilzomib	FDA approval in 2012	epoxyketones	irreversible	CT-L	IV
Oprozomib	currently in clinical trials	epoxyketones	irreversible	CT-L	Oral
Ixazomib	FDA approval in 2015	boronic acid	reversible	CT-L	IV, Oral
Delanzomib	currently in clinical trials	boronic acid	reversible	CT-L	IV, Oral
Marizomib	currently in clinical trials	salinosporamide	irreversible	T-L, CT-L, C-L	IV, Oral

T-L, trypsin-like activity; CT-L, chymotrypsin-like activity; C-L, caspase-like activity.

## Data Availability

Not applicable.
